# Long-Term Morphological Changes of Symptomatic Lacunar Infarcts and Surrounding White Matter on Structural Magnetic Resonance Imaging

**DOI:** 10.1161/STROKEAHA.117.020495

**Published:** 2018-03-22

**Authors:** Caroline M.J. Loos, Stephen D.J. Makin, Julie Staals, Martin S. Dennis, Robert J. van Oostenbrugge, Joanna M. Wardlaw

**Affiliations:** 1 ^1^From the Cardiovascular Research Institute Maastricht, Maastricht University Medical Centre, Maastricht University, the Netherlands (C.M.J.L., J.S., R.J.v.O.); 2 ^2^Department of Neurology, Maastricht University Medical Centre, Maastricht University, the Netherlands (C.M.J.L., J.S., R.J.v.O.); 3 ^3^Brain Research Imaging Centre, Neuroimaging Sciences, Centre for Clinical Brain Sciences, University of Edinburgh, Scotland, United Kingdom (S.D.J.M., M.S.D., J.M.W.); 4 ^4^UK Dementia Research Institute, University of Edinburgh, Scotland, United Kingdom (J.M.W.); 5 ^5^Scottish Imaging Network, A Platform for Scientific Excellence Collaboration, Scotland, United Kingdom (J.M.W.); 6 ^6^Department of Neurology, Universitair Ziekenhuis Antwerpen, Edegem, Belgium (C.M.J.L.).

**Keywords:** cerebral small vessel diseases, follow-up studies, stroke, lacunar, Wallerian degeneration

## Abstract

**Background and Purpose—:**

Insights into evolution of cerebral small vessel disease on neuroimaging might advance knowledge of the natural disease course. Data on evolution of sporadic symptomatic lacunar infarcts are limited. We investigated long-term changes of symptomatic lacunar infarcts and surrounding white matter on structural magnetic resonance imaging.

**Methods—:**

From 2 nonoverlapping, single-center, prospective observational stroke studies, we selected patients presenting with lacunar stroke symptoms with a recent small subcortical (lacunar) infarct on baseline structural magnetic resonance imaging and with follow-up magnetic resonance imaging available at 1 to 5 years. We assessed changes in imaging characteristics of symptomatic lacunar infarcts and surrounding white matter.

**Results—:**

We included 79 patients of whom 32 (41%) had complete and 40 (51%) had partial cavitation of the index lesion at median follow-up of 403 (range, 315–1781) days. In 42 of 79 (53%) patients, we observed a new white matter hyperintensity adjacent to the index infarct, either superior (white matter hyperintensity cap, n=17), inferior (white matter hyperintensity track, n=13), or both (n=12).

**Conclusions—:**

Half of the sporadic symptomatic lacunar infarcts developed secondary changes in superior and inferior white matter. These white matter hyperintensity caps and tracks may reflect another aspect of cerebral small vessel-related disease progression. The clinical and prognostic values remain to be determined.

## Abstract 2

Supplemental Digital Content is available in the text.

Cerebral small vessel disease (cSVD) is a pathological process involving small perforating vessels in the brain.^1^ It may cause symptomatic lacunar infarcts but also silent brain damage that is visible on structural magnetic resonance imaging (MRI), including white matter hyperintensities (WMHs) and lacunes.^2^ Insights into the radiological evolution of cSVD features are important because they might increase our knowledge of vascular disease and neurodegeneration and could be used as surrogate markers for therapeutic studies.

However, there is limited information on long-term tissue damage in sporadic cSVD. Although central cavitation (lacune formation) of the sporadic symptomatic lacunar infarct has been reported in several studies,^3–6^ only 1 study investigated long-term perilesion morphological changes of sporadic symptomatic lacunar infarcts, finding new WMH (caps) lateral or superior to 18% (15 of 82) of sporadic symptomatic lacunar infarcts.^7^ Other studies have suggested that sporadic symptomatic infarcts are associated with widespread secondary degeneration in adjacent white matter tracts.^8^

In contrast, in patients with cerebral autosomal dominant arteriopathy with subcortical infarcts and leukoencephalopathy (CADASIL)—a genetic cSVD—several studies document long-term perilesional and remote morphological changes of cSVD lesions, including development of new lacunes at the edge of WMH or spread of WMH around incident lacunes.^9,10^

The aim of the present study was to investigate further the natural disease course of sporadic cSVD by assessing the long-term, morphological lesional and perilesional changes in symptomatic lacunar infarcts and adjacent white matter on structural MRI.

## Materials and Methods

The data that support the findings of this study are available from the corresponding author on reasonable request.

### Study Population and Recruitment

We used longitudinal data from 2 nonoverlapping, prospective observational studies in patients with minor (ie, nondisabling) ischemic stroke who were all scanned on 1 research-dedicated MRI scanner (MSS [Mild Stroke Study]-1, 2003–2007; MSS-2, 2010–2012). The design of both studies has been described before.^11–13^ Briefly, both studies recruited patients who presented with minor ischemic stroke, including symptoms of lacunar stroke, to the regional stroke service. Patients with contraindications to MRI and unstable medical conditions were excluded. For the present study, we selected all patients with (1) a clinical diagnosis of lacunar stroke and a relevant (to symptoms) acute small subcortical infarct (lacunar infarct; index lesion) on diffusion MRI at presentation and (2) a follow-up structural MRI between 1 and 5 years after the index stroke. We recorded age at stroke onset, sex, and vascular risk factors, as defined earlier.^11^ Both studies were approved by the Scotland and Lothian Research Ethics Committee, and all patients gave written informed consent.

### MR Imaging

All patients had a brain MRI at baseline (median, 4; range, 0–57; days after stroke onset) on a research-dedicated 1.5 Tesla MR scanner (Signa LX; General Electric, Milwaukee, WI). Sequences included axial diffusion-weighted imaging, fluid-attenuated inversion recovery, T2-weighted and T2*-imaging, and sagittal T1-weighted sequences (details were described elsewhere^11,13^). An experienced neuroradiologist (J.M.W.) assessed the MR images for presence of the acute small subcortical (lacunar) infarct, cSVD lesions, and brain atrophy, all according to the STRIVE criteria (Standards for Reporting Vascular Changes on Neuroimaging).^2^ We defined an acute small subcortical (lacunar) infarct as a round or ovoid (axial diameter, <20 mm) lesion in the basal ganglia, internal capsule, centrum semiovale, or brain stem, which was hyperintense on diffusion-weighted imaging, had reduced signal on apparent diffusion coefficient imaging, with or without increased signal on fluid-attenuated inversion recovery or T2-weighted imaging.^2^ We refer to this symptomatic acute lesion as the index lacunar lesion. The diameter of each index lacunar infarct was measured in 3 directions on fluid-attenuated inversion recovery (we report maximum diameter). Baseline MRI was also rated separately and blind to the index lesion progression for presence of lacunes (number and location), WMH (periventricular and deep; Fazekas score^14^), cerebral microbleeds (location and number; modified brain observer microbleed scale^15^), and perivascular spaces (5-point score in basal ganglia and centrum semiovale separately^11^).

We selected those patients who had a follow-up brain MRI at 1 to 5 years after index stroke (median, 403; range, 315–1781; days after index stroke) as part of the original studies (MSS-1 patients had a follow-up MRI at 3 years after stroke and MSS-2 patients had a follow-up MRI at 1 year after stroke; some had longer intervals). The follow-up MR protocol was the same as the baseline MR protocol and performed at the same MR scanner. We visually scored the follow-up appearance of the index lacunar lesion and adjacent white matter on MRI, blind to the other cSVD features, by examining the index lesion and at least 3 MRI slices superior (ie, in the direction of the cortex) and 3 MRI slices inferior (ie, in the direction of the brain stem) from the index lacunar infarct. We assessed the appearance of the index lacunar lesion itself by measuring the diameter in 3 planes as at baseline (we report on maximum diameter) and the appearance, including different degrees of cavitation (no cavitation, partial cavitation, or complete cavitation), as defined previously.^3,5^ Definitions and imaging characteristics of different degrees of cavitation are in Methods in the online-only Data Supplement.

**Figure 1.**
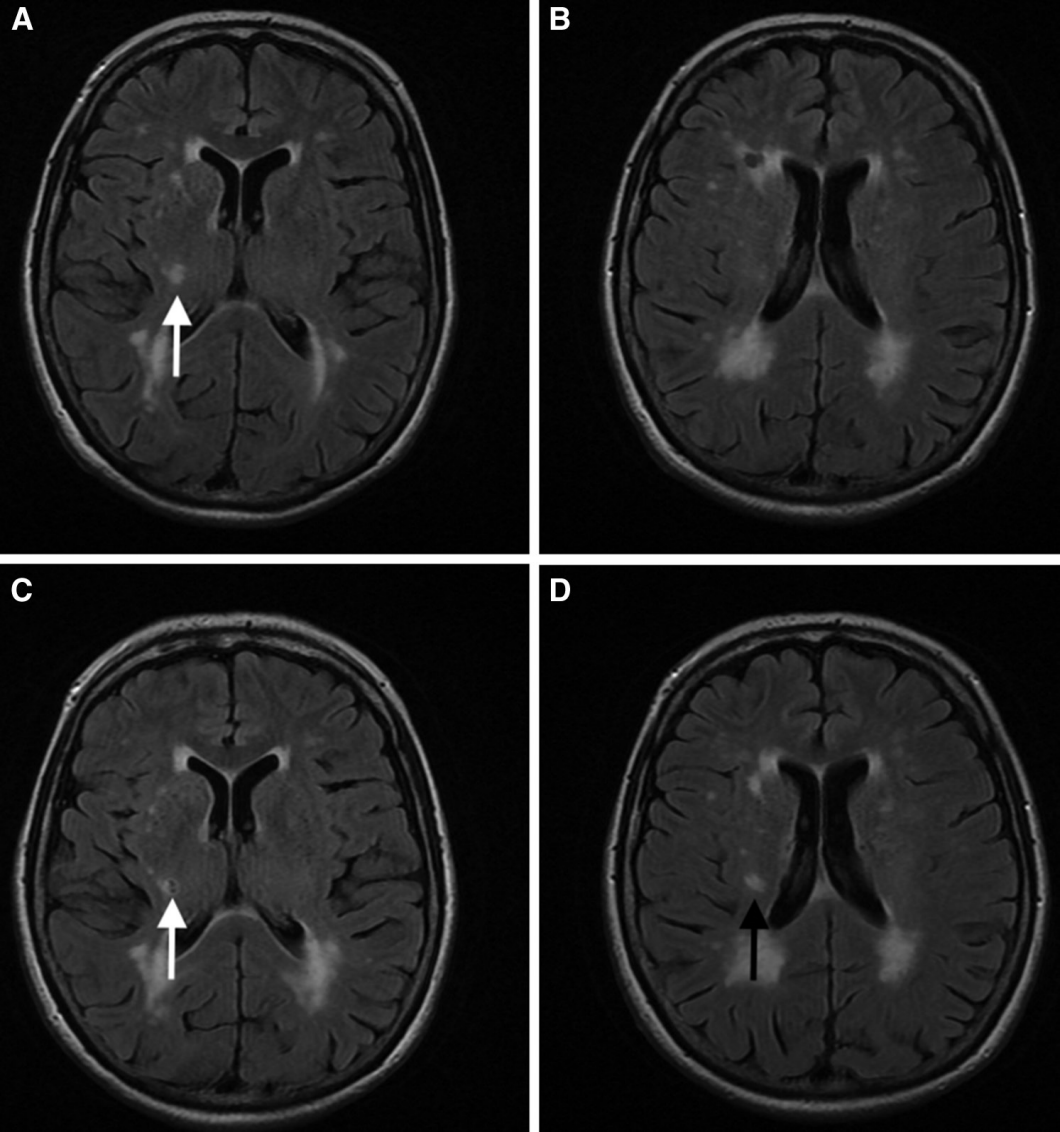
The occurrence of a white matter hyperintensity (WMH) cap adjacent to index lacunar lesion. **A**, Baseline magnetic resonance imaging (MRI), performed 1 d after stroke onset, sporadic symptomatic lacunar infarct in the right internal capsule (white arrow; fluid-attenuated inversion recovery [FLAIR]). **B**, Baseline FLAIR image on MRI slice superior to the index lesion. **C**, Follow-up MRI at 1 y (353 d) after index stroke, partial cavitated lesion (lacey-like appearance) on FLAIR (white arrow). **D**, Follow-up FLAIR image on MRI slice superior to the cavitated index lesion, showing a WMH cap adjacent to the index lacunar lesion (black arrow).

**Figure 2.**
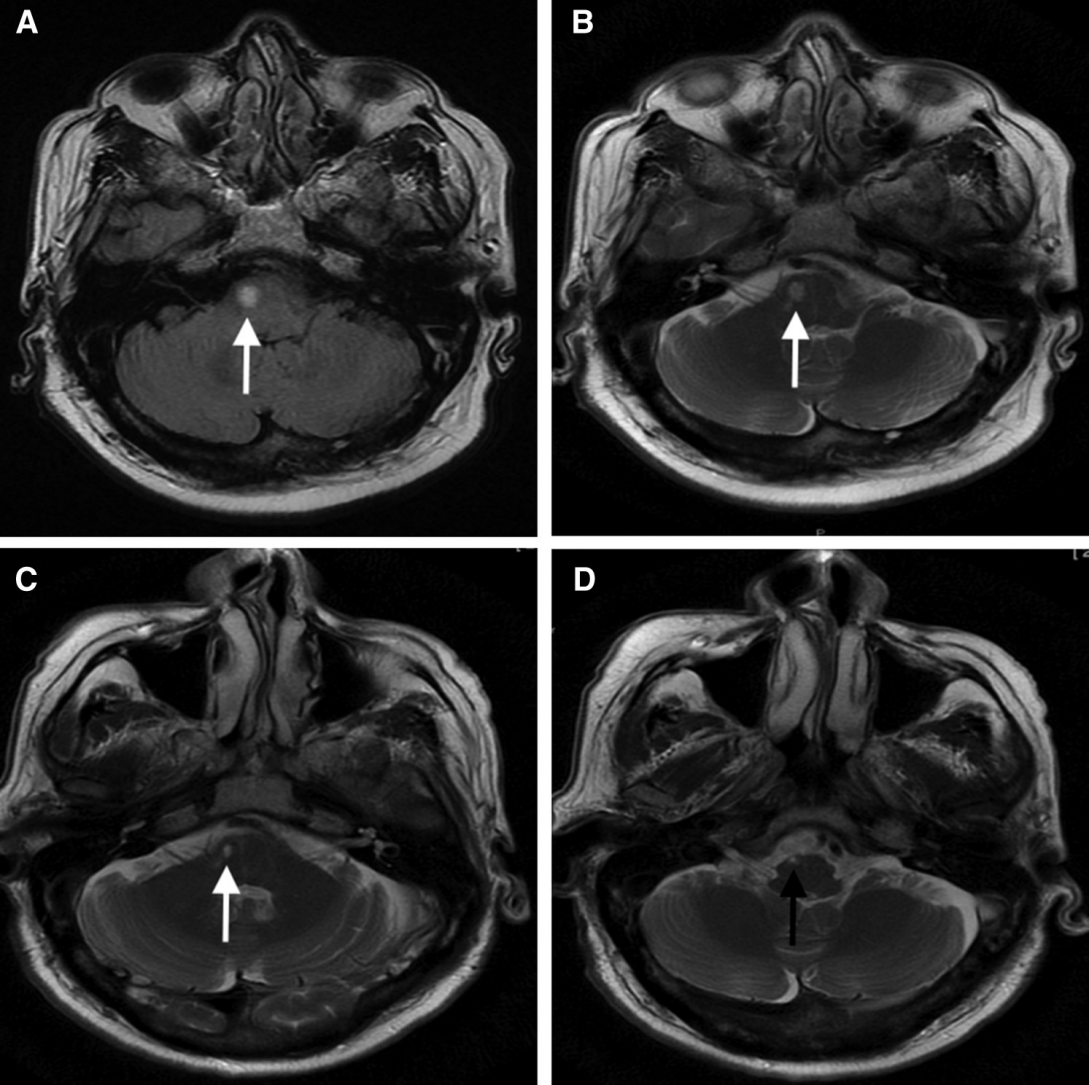
The occurrence of a white matter hyperintensity (WMH) track adjacent to index lacunar lesion. **A** and **B**, Baseline magnetic resonance imaging (MRI), performed 11 d after stroke onset, sporadic symptomatic lacunar infarct in the right pons (white arrow; fluid-attenuated inversion recovery; T2-weighted imaging). **C**, Follow-up MRI at 1 y (412 d) after index stroke, cavitated lacunar lesion in the right pons on T2-weighted imaging (white arrow). **D**, Follow-up MRI, WMH track proximal to index lacunar lesion in the right pons, visible on >2 MRI slices inferior to the index lacunar lesion (black arrow; T2-weighted imaging).

Inferior and superior from index lacunar lesion, we assessed the presence of new WMH (WMH track and WMH cap, respectively). Figure 1 shows an example of a WMH cap adjacent (superior) to the index lesion. These WMH caps were single, mostly round or ovoid with indistinct margins, were not visible at baseline, and could be clearly outlined from diffuse WMH (if present). Figure 2 shows an example of a WMH track, located inferior from the index lacunar infarct. WMH tracks were round and small (often narrower than caps), were elongated (visible ≥2 MRI slices inferior to the index lacunar lesion), and followed the descending white matter tract, similar to Wallerian degeneration that is common after larger territorial infarcts. WMH tracks could be clearly outlined from extensive WMH (if present) and were not present at baseline. WMH progression was assessed by using a validated visual WMH change scale (modified Rotterdam progression scale^16^), not including the WMH cap or track in this assessment.

### Statistical Analysis

Data differences between groups were tested using independent samples *t* test (normally distributed variables), Mann–Whitney *U* test (nonparametric data), and Pearson χ^2^ and Fisher test (categorical variables). Associations with occurrence of WMH caps and tracks were tested by univariable logistic regression analysis (including age, sex, vascular risk factors [hypertension, smoking, diabetes mellitus, and hypercholesterolemia], baseline MRI features [extensive basal ganglia perivascular spaces (grade 2–4), extensive WMH (Fazekas periventricular grade 3 or deep grade 2 or 3)], and follow-up MRI features [time between index stroke and follow-up MRI, and WMH progression], which are known variables associated with the presence and progression of WMH). Statistical significance was set at *P*<0.05 (2-tailed). Analyses were performed using SPSS statistical software package (SPSS, version 23.0; SPSS, Inc, Chicago, IL).

## Results

**Figure 3.**
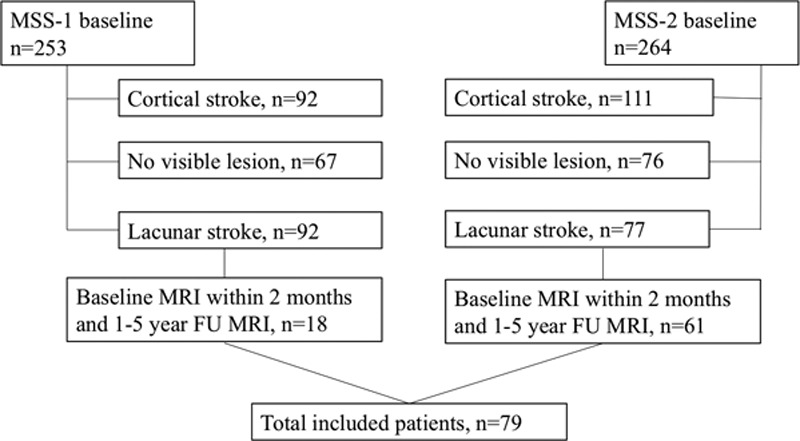
Patients’ recruitment characteristics. FU indicates follow-up; MRI, magnetic resonance imaging; and MSS, Mild Stroke Study.

Of 517 patients with stroke in the original studies, 169 (33%) had a lacunar stroke with an acute symptomatic (index) small subcortical (lacunar) infarct on MRI. Of these, 79 patients had a 1- to 5-year follow-up MRI and met the inclusion criteria for the present study. Recruitment details are shown in Figure 3. Table 1 shows baseline patient characteristics. The majority of patients were first ever strokes. Five (6%) patients had a history of transient ischemic attack, 5 (6%) had a history of an ischemic stroke, and 3 (4%) had both. Five (6%) patients had a recurrent stroke during follow-up: 3 were lacunar stroke (all in the contralateral hemisphere) and 2 were cortical stroke (1 in the ipsilateral hemisphere). Twenty-two (28%) patients had WMH progression (≥1 point on Rotterdam progression scale).

**Table 1.**
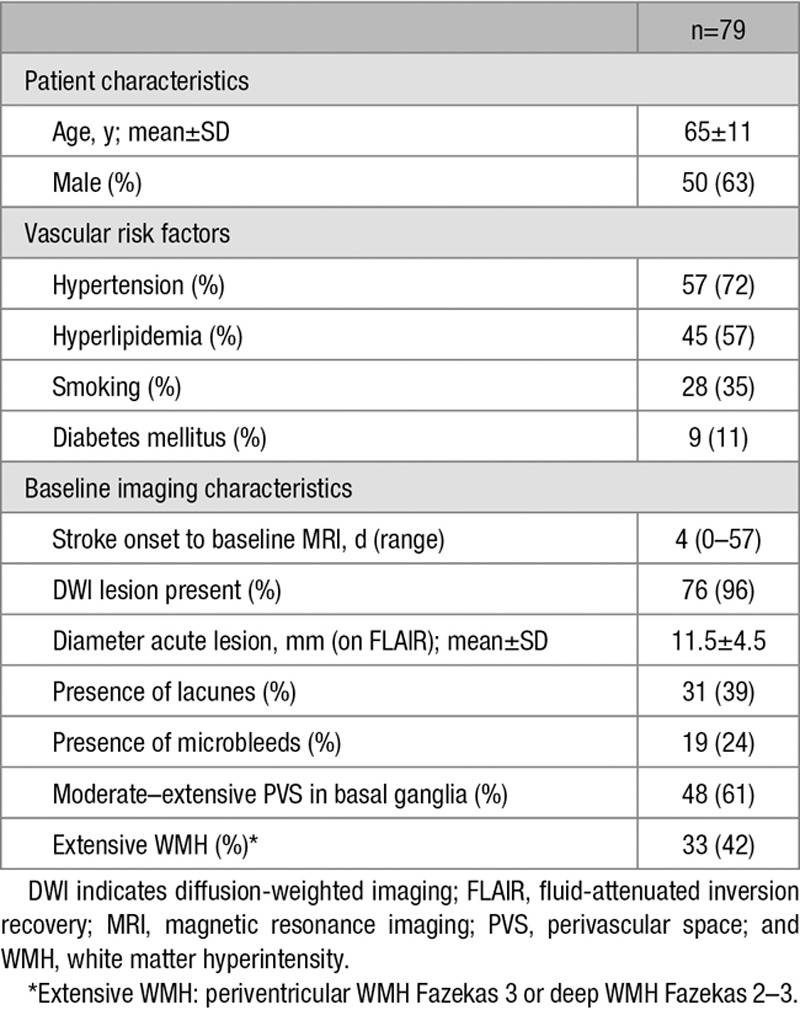
Baseline Characteristics

### Evolution of the Index Lacunar Infarct on Follow-Up Imaging

On follow-up imaging, some degree of cavitation was seen in 72 of 79 (91%) index lacunar infarcts, partial in 40 of 79 (51%), and complete in 32 of 79 (41%) index lacunar infarcts. Five of 79 (6%) index lesions had disappeared during follow-up, although one of these had developed a WMH cap (below). Two (3%) index lacunar lesions resembled a noncavitated WMH on follow-up imaging. No patient-related or imaging-related variables were associated with any degree of cavitation.

### WMH Adjacent to Index Lacunar Infarct on Follow-Up Imaging

We observed a new WMH adjacent to the index lacunar infarct in 42 of 79 (53%) patients: in 17 of 79 (22%) patients, the new WMH was superior (WMH cap); in 13 of 79 (16%) patients, the new WMH was inferior (WMH track); and in 12 of 79 (15%) patients, it was both superior and inferior from the index lacunar infarct. Table 2 shows characteristics of patients with WMH caps and tracks. WMH caps were most frequent on index lesions in the centrum semiovale (62%) but were also present in the internal and external capsule or nucleus lentiformis (24%), thalamus (10%), and brain stem (4%). We observed WMH tracks mostly in the centrum semiovale (64%) but also in the brain stem (16%), in the internal and external capsule and nucleus lentiformis (20%).

**Table 2.**
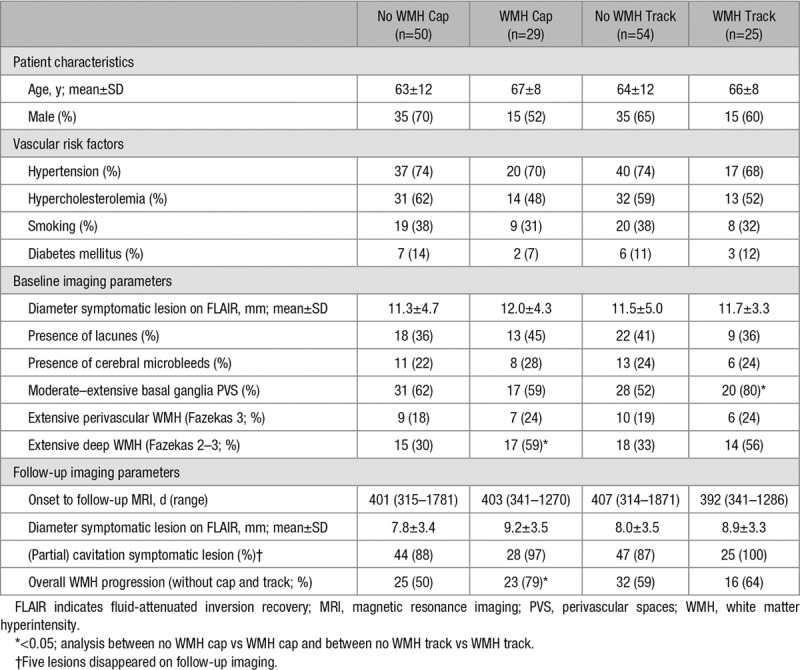
Occurrence of WMH Caps and Tracks Adjacent to Index Lacunar Lesions

WMH caps were associated with presence of extensive deep WMH at baseline (odds ratio, 3.31; 95% confidence interval, 1.27–8.59; *P*<0.05) and with overall WMH progression (odds ratio, 3.83; 95% confidence interval, 1.33–11.02; *P*<0.05). WMH tracks were associated with moderate–extensive basal ganglia perivascular spaces at baseline (odds ratio, 3.71; 95% confidence interval, 1.22–11.34; *P*<0.05). No other patient-related or imaging-related variables, including index lacunar infarct cavitation and time between baseline to follow-up imaging, were associated with occurrence of WMH caps or tracks.

## Discussion

We assessed the long-term radiological evolution of sporadic symptomatic lacunar infarcts and perilesional white matter tissue on structural MRI. We demonstrate that, during follow-up of between 1 and 5 years, more than half of these sporadic symptomatic lacunar infarcts showed secondary perilesional morphological changes (WMH caps and tracks).

WMH caps were associated with baseline deep WMH and WMH progression. Because severe WMH at baseline is the strongest predictor of WMH progression, similar risk factors might also be linked to the development of these WMH caps, although we could not find any association with conventional risk factors, such as age and hypertension. The present study confirms the results of a prior longitudinal MRI study that reported new WMH caps superior from sporadic symptomatic lacunar infarcts in 15 of 82 patients during 2-year follow-up.^7^

The new WMH tracks that appeared inferior from the index lacunar lesion seemed to be following a descending white matter tract similar to Wallerian degeneration. Wallerian degeneration of descending tracts is a well-known phenomenon after territorial stroke and reflects a pathological process with disintegration of axons, macrophage infiltration, degradation of myelin, and finally gliosis and atrophy of the affected tracts.^17,18^ Although we found that many index lacunar infarcts also underwent some degree of central cavitation, there was no association between progressive perilesional (WMH caps or tracks) and central lesion tissue damage.

We suggest that secondary changes in white matter surrounding a sporadic index lacunar infarct should be considered a separate MRI feature of worsening brain damage and so disease progression. Their possible clinical and prognostic value requires further study. Wallerian degeneration has been associated with poor motor function recovery after territorial stroke.^19^ Longitudinal MRI studies with tractography have shown that subcortical infarcts in patients with CADASIL cause focal thinning in the remote cortex by degeneration of connected white matter tracts.^9,20^ Further, diffusion tensor imaging studies have shown secondary tract degeneration in white matter tracts, remote from sporadic symptomatic lacunar infarcts, the severity of which was independently related to worse cognitive functioning.^8^

Several studies have assessed long-term cavitation of sporadic symptomatic lacunar infarcts.^3–6^ As in 2 former studies,^5,6^ we found a high partial or complete cavitation rate. Although some of the apparent difference in cavitation rates between studies likely reflects differences in definitions or interpretation of cavitation, the formation of even a partial cavity over time could reflect more interruption to white matter connectivity and might lead to worse clinical outcome. Therefore, it could be worthwhile to compare connectivity of white matter tracts (with diffusion tensor imaging) in cavitated versus noncavitated lesions. Some symptomatic lacunar lesions disappear, at least macroscopically, which could imply that conventional MRI underestimates the total cSVD-related brain damage.

The main strengths of our study are a relatively long follow-up time, standardized MRI protocols, and use of 1 carefully monitored MRI scanner. We used standardized international consensus criteria^2^ to describe cSVD imaging findings, and all assessments were blinded. However, our study also has limitations. Although this is one of the largest neuroimaging follow-up studies on this topic, our sample is relatively small for a common disease like lacunar stroke. The original studies only included patients with a (nondisabling) stroke, and for the present analysis, we selected patients with a follow-up MRI at 1 to 5 years after stroke for the original studies, both of which could have introduced bias. A few patients had clinically indicated MRI scans, which could also have led to a potential selection bias and affected the time to follow-up scanning. Three patients did not have a positive diffusion-weighted imaging, which could have caused that the wrong lesion was counted for the symptomatic lacunar infarct. However, we only included patients with a definitive clinical diagnosis of lacunar stroke, assessed by a panel of stroke experts, and the lesion had to be compatible with clinical signs. Our study describes imaging findings and is too small to correlate these with clinical features like cognition or stroke recovery. Further, we acknowledge the lack of statistical power to identify possible predictors of WMH caps and tracks. Larger prospective studies in an independent cohort are necessary to confirm our findings.

## Summary

In conclusion, many sporadic symptomatic lacunar infarcts developed secondary changes in the adjacent inferior or superior white matter during follow-up and showed some degree of cavitation over time. Adjacent WMH caps and tracks may reflect another aspect of cSVD-related disease progression and neurodegeneration, with possible clinical and prognostic value. Larger prospective studies are necessary to confirm this hypothesis.

## Acknowledgments

We thank the patients, their families, and the staff of the Brain Research Imaging Centre, Edinburgh, where magnetic resonance imaging scanning was performed.

## Sources of Funding

The contributing studies were funded by the Chief Scientist Office of the Scottish Executive (grant 217 NTU R37933), the Wellcome Trust (grants 075611 and WT088134/Z/09/A), and Row Fogo Charitable Trust. The imaging was performed at the Brain Research Imaging Centre Edinburgh, which is supported by the SINAPSE (Scottish Imaging Network, A Platform for Scientific Excellence) collaboration and the Chief Scientist Office of the Scottish Government (http://www.bric.ed.ac.uk/). This work was supported by European Union Horizon 2020 (EU H2020), PHC- 03 to 15, project No. 666881, SVDs@Target, and the Fondation Leducq Transatlantic Network of Excellence for Study of Perivascular Spaces in Small Vessel Disease, ref No. 16 CVD 05. Dr Loos was supported by the Dutch Alzheimer Foundation.

## Disclosures

None.

## Supplementary Material
