# Supplementary material for: Long-Term Morphological Changes of Symptomatic Lacunar Infarcts and Surrounding White Matter on Structural Magnetic Resonance Imaging
Source: Stroke. 2018 Mar 22;49(5):1183–8. doi: 10.1161/STROKEAHA.117.020495 (PMC5916475; doi:10.1161/STROKEAHA.117.020495)
Supplement: Supplementary file 1 [file str-49-1183-s001.pdf]

SUPPLEMENTAL MATERIAL

Long-term morphological changes of symptomatic lacunar infarcts and surrounding white matter on structural MRI.

Caroline M.J. Loos[1], MD; Stephen D.J. Makin[2], MRCP; Julie Staals[1], MD, PhD; Martin S. Dennis[2], MD; Robert J. van Oostenbrugge[1] MD, PhD; Joanna M. Wardlaw[2-4,] MD, FRSE, FMedSci.

1. Department of Neurology and Cardiovascular Research Institute Maastricht (CARIM), Maastricht University Medical Centre (MUMC+), University Maastricht, the Netherlands. Department of Neurology, MUMC, PO Box 5800, 6202AZ Maastricht, The Netherlands.

2. Brain Research Imaging Centre, Neuroimaging Sciences, Centre for Clinical Brain Sciences (CCBS) FU303e, The University of Edinburgh, Chancellor's Building, 49 Little France Crescent, Edinburgh, EH16 4SB, UK.

3. Scottish Imaging Network, A Platform for Scientific Excellence (SINAPSE) Collaboration.

4. UK Dementia Research Institute at The University of Edinburgh. Dementia Research Institute, c/o The Medical Research Council. One Kemble Street. London WC2B 4ANUK, UK.

Correspondence to Joanna M. Wardlaw, Centre for Clinical Brain Sciences (CCBS) FU303e, The University of Edinburgh, Chancellor's Building, 49 Little France Crescent, Edinburgh, EH16 4SB, UK. Phone: +44 (0)131 465 9599. Fax: +44 131 332 5150. E-mail: Joanna.wardlaw@ed.ac.uk.

Cover title: Morphological changes of lacunar infarcts.

Supplemental Figure 1.

Supplemental Methods

Assessment of vascular risk factors.

In the primary studies[1-2], experienced physicians qualified in stroke medicine recorded baseline demographics, vascular risk factors, and other details, including current smoking. Hypertension (blood pressure > 140/90 mm Hg), diabetes mellitus (fasting blood glucose > 6.1 mmol/L) and hypercholesterolemia (total cholesterol > 5.0 mmol/L) were defined as previously diagnosed by a physician/general practitioner, on current treatment, or newly diagnosed at stroke presentation.

Imaging characteristics

All patients underwent a brain MRI scan on the same 1.5 Tesla MRI scanner (Signa LX; General Electric, Milwaukee, WI) operating in research mode, and using a self-shielding gradient set with maxi- mum strength of 33 mT/m, and an 8-channel phased-array head coil. The scanner was operated within a tight quality assurance program to maintain uniform performance. Sequences included axial DWI (30-direction axial diffusion tensor imaging, b=1000 s/mm2 and 2×b0 acquisitions, repetition time/echo time [TR/TE]=7700/82 ms, 24×24 cm eld of view [FoV], 128×128 acquisition matrix, 28×5-mm slices), T2-weighted (TR/TE=6000/90 ms, 24×24 cm FoV, 384×384 propeller acquisition, 1.5 averages), axial fluid-attenuated inversion recovery (TR/TE/inversion time =9000/153/2200, 24×24 cm FoV, 384 (anterior-posterior)×224 acquisition matrix), T2* (TR/ TE=800/15 ms, 20° ip angle, 24 (anterior-posterior)×18 cm FoV, 384×168 acquisition matrix, 2 averages, all with 28×5-mm slices and 1-mm slice gap), and sagittal 3D T1-weighted (TR/TE/inversion time=7.3/2.9/500 ms, 8° ip angle, 330 (superior-inferior)×214.5 cm FoV, 256×146 acquisition matrix, 100×1.8-mm slices).

Cavitation on MRI

We visually assessed the appearance of the index lacunar lesion, including different degrees of cavitation. Figure I shows the imaging characteristics of different degrees of cavitation.

Absent cavitation was defined as a lesion without any evidence of cavitation. Partial cavitation was defined as a lesion with one of the following characteristics: a lacey-like or spongiform appearance with areas of marked hypointensity in the core of the lesion on FLAIR; a lesion with a core with equivalent to cerebrospinal fluid (CSF) signal on T2, but which is not visible on FLAIR; a lesion with a core with equivalent to CSF signal on T2, but with presence of a hypointensity, not as dark as CSF, on FLAIR; a lesion with a core with equivalent to CSF signal on T2, but with presence of a non-cavitated WMH on FLAIR; and a lesion were only a small core in part of the lesion is visible, both on T2 and FLAIR. Complete cavitation (lacune) was defined as a cavity with signal intensity comparable to CSF on FLAIR and T2, with a thin surrounding hyperintense halo (gliosis) on FLAIR.

Figure I Imaging characteristics of different degrees of cavitation.

MRI feature	Imaging characteristics	T2	FLAIR
No cavitation	Hyperintense on FLAIR and T2 imaging, without any evidence of cavitation	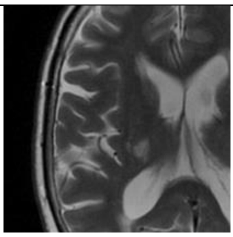	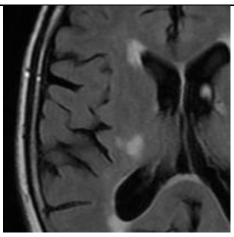
Partial cavitation			
lacey-like FLAIR	Lacey-like appearance with areas of marked hypo-intensity in the core (often with septa) on FLAIR, and a core with the equivalent to CSF signal on T2 imaging	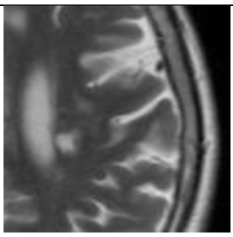	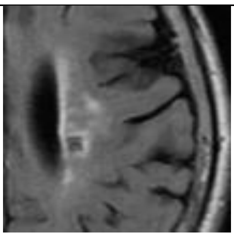
T2 hole like, FLAIR not visible	Lesion has a core with the equivalent to CSF signal on T2, but is not visible on FLAIR imaging	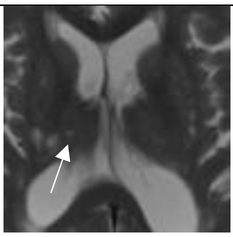	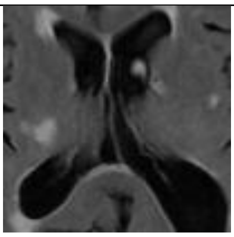
T2 hole like, FLAIR not as dark as CSF	Lesion has a core with the equivalent to CSF signal on T2, and a hypointensity in the core, which is not as dark as CSF, on FLAIR imaging	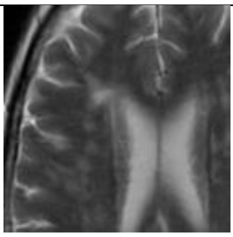	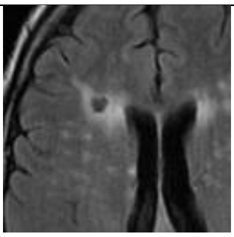
T2 hole like, FLAIR WMH	Lesion has a core with the equivalent to CSF signal on T2, and resembles a (non-cavitated) WMH on FLAIR imaging	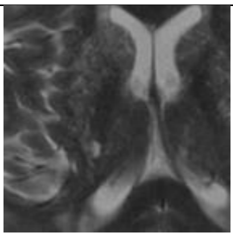	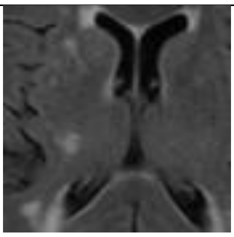
small hole in part of lesion both T2 and FLAIR	Only a small core in part of the lesion is cavitated (with the equivalent to CSF signal) on T2 and FLAIR imaging	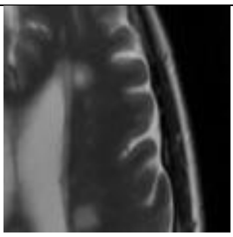	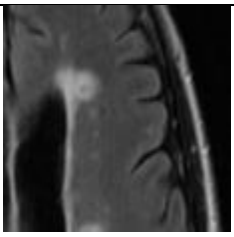

Complete cavitation	Hyperintense (equivalent to CSF signal) core on T2, with hypointense signal intensity comparable to CSF and with a thin surrounding hyperintense halo (gliosis) on FLAIR imaging	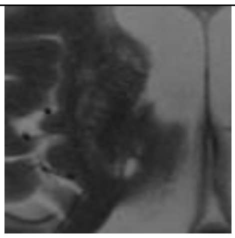	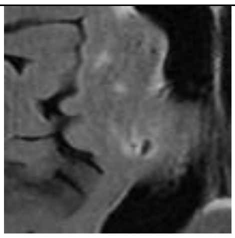

WMH: white matter hyperintensity; CSF: cerebrospinal fluid

References

1. Doubal FN, MacLullich AM, Ferguson KJ, Dennis MS, Wardlaw JM. Enlarged perivascular spaces on MRI are a feature of cerebral small vessel disease. Stroke. 2010;41:450–454.

2. Valdés Hernández Mdel C, Armitage PA, Thrippleton MJ, Chappell F, Sandeman E, Muñoz Maniega S, et al. Rationale, design and methodology of the image analysis protocol for studies of patients with cerebral small vessel disease and mild stroke. Brain Behav. 2015;5:e00415.
